# Magnetic and Optical Properties of Submicron-Size Hollow Spheres

**DOI:** 10.3390/ma3021244

**Published:** 2010-02-21

**Authors:** Quan-Lin Ye, Hirofumi Yoshikawa, Kunio Awaga

**Affiliations:** Research Center for Materials Science & Department of Chemistry, Nagoya University, Furo-cho, Chikusa-ku, Nagoya 464-8602, Japan; E-Mail: yoshikawah@mbox.chem.nagoya-u.ac.jp (H.Y.)

**Keywords:** hollow sphere, cobalt and cobalt oxides, iron and iron oxides, antiferromagnet, magnetic hysteresis, domain structure, magnetization relaxation, exchange bias, uncompensated spin, Mie scattering

## Abstract

Magnetic hollow spheres with a controlled diameter and shell thickness have emerged as an important class of magnetic nanomaterials. The confined hollow geometry and pronouncedly curved surfaces induce unique physical properties different from those of flat thin films and solid counterparts. In this paper, we focus on recent progress on submicron-size spherical hollow magnets (e.g., cobalt- and iron-based materials), and discuss the effects of the hollow shape and the submicron size on magnetic and optical properties.

## 1. Introduction

Nanostructured magnetic materials have attracted tremendous attention because of their potential for clarifying fundmental nanomagnetism and their technological applications in diverse fields [1,2,3,4,5,6,7], including ultra-high-density data storage, spintronics, ferrofluids, magneto-optical devices, and biomedicine. To date, magnetic nanostructures have been produced in a variety of geometries and dimensions, such as nanospheres/dots [3], nanoboxes [4], nanorings [5], nanowires/rods/tubes [6], and thin films [7]. The magnetic properties of nanostructured materials are usually governed by their shape, size, and material parameters. For example, as the material size decreases, the domain structure changes from a multidomain to a single-domain structure due to a competition between magnetostatic energy and domain-wall energy; the coercive force increases monotonically with a decrease in the diameter of spherical particles. Below a critical dimension, a transition between the single-domain state and vortex state has been observed in many magnetic nanostructures [2,8]. 

Among various morphologies of nanomaterials, hollow spheres are of great interest because of their high ratio of surface to volume, large pore volume and low density, which could be exploited for promising applications in controlled encapsulation-release of drugs and medical diagnostics [9,10], energy storage and conversion [11,12], photocatalysis [12,13], chemical sensors [14,15], and photonic crystals [16]. For instance, the magnetite hollow nanocapsules developed by Hyeon group have been utilized as mutifunctional nanocarriers to deliver drugs and provide contrast for magnetic resonance imaging [9]. Porous hollow Fe_3_O_4_ nanoparticles have been used for targeted delivery and controlled release of cisplatin, which is a powerful therapeutic agent against numerous solid tumors [10]. In addition, the hollow structure contributes another parameter by which the physical properties can be tuned. By changing the size of the hollow core and shell thickness, tunable plasmon resonances have been realized in Au hollow nanostructures, making them appropriate for use as optical imaging contrast agents [17]. 

In the context of magnetism, magnetic hollow spheres can show unique physical properties compared to those of flat thin films and their solid counterparts of the same sizes, due to their confined hollow geometry and pronoucedly curved surfaces [18,19,20,21,22,23,24,25,26]. It is known that coercivity is dependent on domain-wall motion and the barrier to domain-wall propagation along a curved surface is larger than that of a flat surface [18]. Theoretical calculations have shown that large domain-wall curvature could lead to the enhancement of coercivity [19]. Therefore, the coercivity can be expected to be higher for curved surfaces. Recently, enhanced coercivity and remanent magnetizations have been found in curved magnetic nanostructures such as macroporous films [18,20,21], nanobowls [22], and hollow spheres [23,24,25,26]. Therefore, assembling nanoparticles into a spherical hollow structure may provide one way to overcome the superparamagnetic limit. A magnetic hollow sphere can maintain single-domain behavior at a much larger diameter than can a solid sphere; the critical single-domain size for FePt hollow spheres with extremely thin shells is more than double of that for the bulk particles [27]. Furthermore, the curved surface may induce plenty of lattice defects, which may provide domain-wall pinning positions [20,21,22] and/or induce weak ferromagnetism in antiferromagnetic materials [28]. 

Over the last decade, much effort has been devoted to studying submicro-/nano-size magnetic hollow spheres of metals (Co [25,29,30], Ni [23,25,31,32,33], and Fe [30]), intermetallic alloys (FePt [34], CoPt [35,36], and NiFe [37]) and metal oxides/ferrites (Co_3_O_4_ [28], CoO [38], Ni [39], α-/γ-Fe_2_O_3_ [40,41,42,43], CoFe_2_O_4_ [44,45,46], and Fe_3_O_4_ [9,10,26,47,48,49,50,51,52,53,54,55,56]). As a result, a number of preparation methods have been developed, such as the template-mediated approach [23,26,28,30,31,32,33,34,36,37,40,43,44,45,46,47,48,49,50,51,52], nanoscale Kirkendall effect [42,54], Ostwald ripening [25,53,55], galvanic replacement [35], and so on. Among them, the template-mediated approach is one of the most prevalent methods, and has been employed by many groups. Templates can be mainly categorized into two types: hard templates (silica particles, polymer beads, and carbon spheres) [23,26,28,30,34,37,40,43,44,45,46,47,48,49] and soft templates (emulsion droplets, supramolecular micelles/vesicles, and gas bubbles) [31,32,33,36,50,51,52]. Soft templates are often used in preparing nanometer-size hollow spheres, since the templates are usually in nanoscale sizes. However, the applicability of this approach has been limited due to the difficulty in controlling the size of the templates and the relatively harsh conditions required to form the templates. Therefore, many hollow spheres, especially with submicron sizes, are prepared by the hard-template method, in which the template particles are coated uniformly with an inorganic material. After removal of the template by etching, dissolution or thermal decomposition, hollow spheres of inorganic material are obtained. Since inorganic metal and metal-oxide materials often exhibit polymorphism, phase-selective products can be easily achieved by optimizing the template-removal conditions [30]. The hard-template approach allows rigorous microstructural control over the synthesized hollow particles, including control of the composition, size distribution and shell thickness.

Although nanoscale magnetic hollow spheres (< 50 nm) have been widely prepared in recent years, and have shown promising applications in biomedical fields because of their superparamagnetic behaviors, it remains important to prepare submicron-size magnetic hollow spheres with highly homogeneous features in terms of size and shape, in order to carry out fundamental magnetic studies, such as investigations of magnetic domain structures and magnetization reversal. The comparatively large size also means that the critical temperatures of magnetic ordering or magnetization blocking would be sufficiently high for practical applications. Further, the submicron size and low-density hollow structure may bring about specific optical response in the visible region. On the other hand, magnetic elements with submicron sizes have shown different physical properties from those with nano- or macro-scale sizes. For example, two characteristic domain states, referred to as “vortex” and “onion” states have been observed in submicron-size ferromagnetic rings, and switching between the two states has been experimentally realized [5]. Submicron-size magnetic elements have also exhibited a facile and controllable manipulation in physical properties which is promising in spintronics, such as Aharonov-Bohm oscillation in a FeNi ring with outer/inner diameter of 500/420 nm [57], and current-driven resonant excitation of magnetic vortices in Permalloy submicron-dots [58]. 

There are many comprehensive reviews of the synthetic strategies and applications of hollow micron-/nanostructures, especially for nonmagnetic materials [9,11,59,60,61,62]. Here we focus on recent research progress on the magnetic hollow spheres with submicronmeter diameters (*i.e.*, about several hundred nanometers). In this paper, we first present a facile synthetic strategy for preparing hollow spheres of different phases, such as ccp-Co, hcp-Co, Co_3_O_4_, α-Fe, Fe_3_O_4_, and α-Fe_2_O_3_. Then we describe unusual physical properties in the submicron-size spherical hollow magnets of ccp-/hcp-Co, Co_3_O_4_ and Fe_3_O_4_, and discuss the effects of the hollow structure and the submicron size. Finally, we provide a conclusion and outlook on future developments in spherical hollow magnets.

## 2. Synthetic Strategy and Characterizations for Spherical Hollow Magnets

As described in the Introduction, the hard template method provides an effective approach to prepare submicron-size hollow spheres with a uniform size distribution; monodisperse polystyrene (PS) beads have been commonly used as templates because they are readily available in a wide range of sizes. Ohnish *et al.* have recently reported various submicron-size magnetic hollow spheres prepared by a homogeneous deposition method using a PS-bead template [30]. As a first step, the PS beads were coated with basic cobalt carbonate (for cobalt-based hollow magnets) or iron hydroxide (for iron-based hollow magnets) by a controlled hydrolysis of urea; gradual heating of an aqueous solution of Co(NO_3_)_2_ (or FeCl_3_), urea, PS, and PVP resulted in an increase in *p*H due to thermal decomposition of urea. As soon as the solution became basic, homogeneous precipitation of basic cobalt carbonate (or iron hydroxides) took place on the surfaces of the PS beads. Aging at 85 °C (or 100 °C) for 24 h allowed inorganic nanocrystals to grow on the surfaces; precursors for cobalt-based (or iron-based) hollow magnets were obtained after centrifugation of the above dispersion and washing with pure water. The optimized conditions are 3.78 mM Co(NO_3_)_2_•6H_2_O, 0.30 wt % PVP, 0.04 M urea, and 0.10 g L^-1^ PS beads at 85 °C for 24 h for cobalt-salt/PS particles; and 1.48 mM FeCl_3_, 22.0 mM urea, 0.10 g L^-1^ PS, 0.90 wt % PVP (K-30), and 0.06 g L^-1^ HCl (36.0 wt %) at 100 °C for 24 h for iron-salt/PS particles, respectively. 

**Figure 1:**
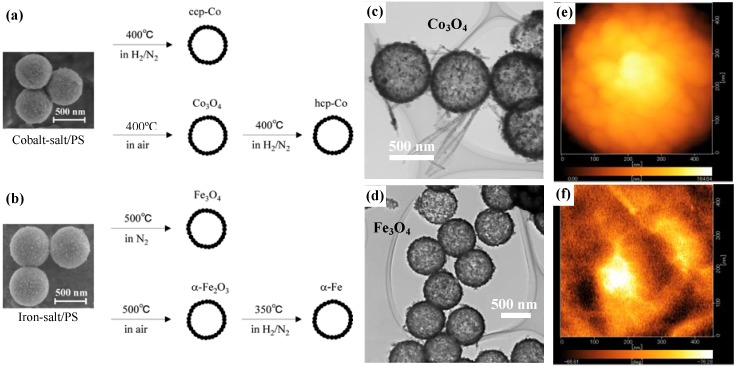
(a) and (b) show the synthetic schemes for preparations of cobalt- and iron-based hollow magnets, respectively; (c) and (d) are typical TEM images for the hollow spheres of Co_3_O_4_ and Fe_3_O_4_, respectively; and (e) and (f) are MFM images of surface morphology and magnetic phase of hcp-Co hollow spheres, respectively.

After calcining the precursors under different atmospheres and temperatures to remove the PS-bead cores, various types (phases) of hollow magnets were obtained, as shown in the schemes in Figure 1(a) and Figure 1(b). The cobalt-salt/PS hybrid particles were calcined in air at 400 °C and were transformed into Co_3_O_4_ hollow spheres. Hcp-Co hollow spheres were obtained by a reduction of the Co_3_O_4_ particles under 1:1 H_2_/N_2_ gas at 400 °C; in contrast, ccp-Co hollow spheres were obtained by the calcination of the parent cobalt-salt/PS particles under H_2_-N_2_ at 400 °C. Hollow spheres of Fe_3_O_4_ and α-Fe_2_O_3_ were selectively prepared by calcination of iron-salt/PS particles at 500 °C in two different atmospheres, namely under N_2_ and in air, respectively. Furthermore, the α-Fe_2_O_3_ particles were reduced to α-Fe under H_2_-N_2_ at 350 °C without any changes in morphology. 

Figure 1(c) and Figure 1(d) respectively show transmission electron microscope (TEM) images for Co_3_O_4_ and Fe_3_O_4_, indicating the formation of hollow structures with a uniform diameter and shell thickness. The mean diameter, shell thickness and average grain size of the various magnetic hollow spheres are summarized in Table 1. Since the grain size is almost half the shell thickness, it appears likely that the shell structure consists of a few layers of crystalline nanoparticles. This is a common feature for all the spherical hollow magnets, as confirmed by the surface morphology observed by magnetic force microscope (MFM) on individual hcp-Co hollow spheres (see Figure 1(e)). Further, the MFM image of the magnetic phase (Figure 1(f)) taken on the top surface of the individual hollow sphere at demagnetization state showed randomly distributed magnetic domains in which each domain covers not only one grain but several grains, confirming the ferromagnetic character of the hcp-Co hollow spheres.

**Table 1:** Size parameters for the spherical hollow magnets.

Chemical formula	Diameter (nm)	Shell thickness (nm)	Grain size (nm)
Co_3_O_4_	360 ± 20	ca. 30	16
Co_3_O_4_	680 ± 10	ca. 40	16
ccp-Co	600 ± 20	ca. 40	14
hcp-Co	630 ± 10	ca. 40	16
Fe_3_O_4_	650 ± 10	ca. 40	25
α-Fe_2_O_3_	630 ± 10	ca. 40	15
α-Fe	500 ± 20	ca. 35	13

## 3. Selective Phases of ccp- and hcp-Co Hollow Spheres

**Figure 2:**
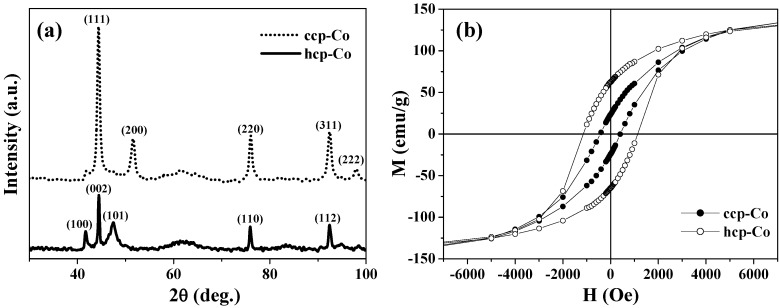
(a) XRD patterns. (b) *M*-*H* curves at 2 K for the ccp- and hcp-Co hollow spheres.

As one of the advantages of the above synthetic strategy, ccp-Co and hcp-Co, which are usually mixed in conventional cobalt samples, were successfully separated in the hollow magnets. The two phases of cobalt hollow magnets were confirmed by the two sets of XRD patterns presented in Figure 2(a), respectively. Figure 2(b) shows the magnetization curves obtained at 2 K for the ccp- and hcp-Co hollow spheres. There is little difference in the saturation magnetization *M*_s_ between them, yet their coercive fields *H*_c_ differ significantly. The *H*_c_ value of 1150 Oe for hcp-Co is much larger than that of 450 Oe for ccp-Co; this finding is consistent with the fact that hcp-Co has greater magnetic anisotropy. Further, the hcp-Co hollow spheres exhibit both enhanced coercivity and a higher remanent ratio (*M*_r_/*M*_s_ = 0.42) than the chains of hcp-Co hollow spheres with a diameter of 500–800 nm (*i.e.*, *H*_c_ = 415 Oe and *M*_r_/*M*_s_ < 0.15) reported by He *et al.* [29]. This result indicates that the magnetization reversal in the present hcp-Co hollow spheres is closer to a coherent rotational mode rather than a nucleation rotational mode, because the crystalline size is similar to the coherence length of cobalt (~15 nm) [29].

Although both the ccp- and hcp-Co hollow spheres were polycrystalline particles, they exhibited great differences in magnetic anisotropy based on their hysteresis behaviors. Recent theoretical calculations on nanosized Co hollow spheres showed that magnetic anisotropy played a significant role in determining the three possible magnetization ground states: the single domain, curling vortex, and three-dimensional onion states [27,63]. Therefore, the ccp- and hcp-Co hollow spheres derived from the same parent precursor particles of cobalt-carbonate/PS may provide a facile system for experimentally investigating the spin configurations and ground states in spherical hollow magnets.

## 4. Uncompensated Magnetizations and Exchange Anisotropy in Co_3_O_4_ Hollow Spheres

Antiferromagnetic (AFM) nanomaterials have recently drawn increasing attention by virtue of their potential for exhibiting quantum tunneling of magnetization, weak ferromagnetism, and exchange anisotropy. Néel suggested that nanoscale AFM nanoparticles can exhibit superparamagnetism and weak ferromagnetism due to the presence of uncompensated magnetization (UCM) on two sublattices [64]. In nanomaterials, UCM arises mainly from the lower coordination of surface atoms, a large surface/volume ratio, and a lack of structural perfection. So far, UCM has been found in various AFM nanomaterials, such as nanoparticles of CoO [65], NiO [66,67], MnO [68], CuO [69] and ferritin [70]. Co_3_O_4_ possesses a normal spinel structure with Co(II) and Co(III) ions respectively locating in the tetrahedral and octahedral interstices of the ccp-lattice of oxide anions and exhibits antiferromagnetism below the Néel temperature of *T*_N_ = 33 K, since the Co(II) ions at the tetrahedral sites form an AFM sublattice with a diamond structure [71]. Recently, significant progress has been achieved in the preparation of nanostructured Co_3_O_4_ materials, such as nanoparticles [72,73] and nanowires [74]. These are promising materials for the induction of enhanced UCM due to their large surface/volume ratios, which automatically lead to the breakings of a number of exchange bonds between the surface atoms.

### 4.1. Uncompensated Magnetization and Its Relaxation

**Figure 3:**
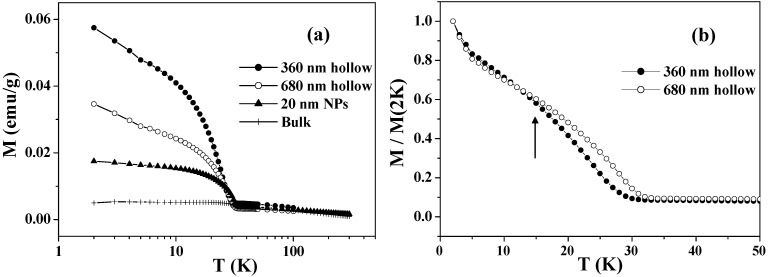
(a) Temperature dependence of FC magnetizations measured in an applied magnetic field of 50 Oe, after cooling down to 2 K with an external field of 50 kOe. (b) Comparison of the normalized FC magnetizations of 360 and 680 nm Co_3_O_4_ hollow spheres.

Yoshikawa *et al.* have reported greatly enhanced field-cooled (FC) magnetizations in submicron-size Co_3_O_4_ hollow spheres [28]. Figure 3(a) shows the temperature dependence of the FC magnetizations in the range of 2 – 300 K for the Co_3_O_4_ hollow spheres with diameters of 360 and 680 nm. Below *T*_N_ = 33 K, they exhibit enhanced magnetizations arising from the UCM. The UCM of the hollow spheres is several times larger than those of bulk samples and nanoparticles of Co_3_O_4_, with characteristic size-dependence: the UCM of the smaller hollow spheres is twice as large as that of the larger ones. This is probably caused by the fact that the increases in the surface/volume ratio and in the number of lattice defects are greater in the smaller hollow spheres. Because the UCMs behaved superparamagnetically, the total magnetic spins became easy to order, and hence the magnetization was increased. The field-induced magnetization below *T_N_* is believed to be stored at the domain boundaries, where the magnetic moments are readily aligned to the field direction, taking advantage of the fact that the long-range AFM correlation is interrupted there. The cooling field energetically favors the domains with a parallel magnetization alignment and induces a remnant magnetization below *T_N_*. Since the Co_3_O_4_ hollow spheres probably involve a number of lattice defects that cause such domains, they exhibit a drastic cooling-field dependence; the stronger the cooling field, the larger FC magnetization below *T*_N_ [28]. Figure 3(b) depicts a comparison between the FC magnetizations of the two samples below 40 K, in which the magnetizations are normalized by the values at 2 K. It can be clearly seen that the UCM of the smaller hollow spheres exhibits a faster relaxation than that of the larger ones as the temperature increases, especially at temperatures over 15 K as indicated by the arrow in Figure 3(b).

**Figure 4:**
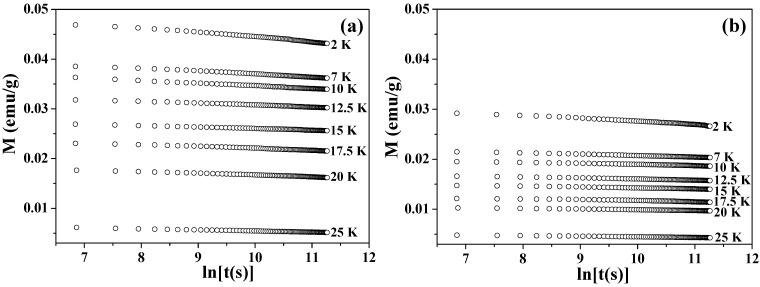
Time dependence of the magnetization (*M* *vs.* ln*t*) at 2–25 K for Co_3_O_4_ hollow spheres of (a) 360 nm and (b) 680 nm.

Figure 4 shows the values of magnetization plotted as a function of time *t* in a logarithmic scale. The time-dependent magnetizations were recorded at each temperature under a zero field, after the samples were cooled down from 300 to 2 K under 50 kOe and then heated up to the target temperatures with a zero field. At each temperature, UCM showed a gradual decrease that was proportional to the logarithm of *t*. This dependence was fitted to the following equation [72,75]

(1) $$M(t)=M(t_{0})(1-S\cdot \text{ln}(t))$$

where $S=(1/M(t_{0}))\cdot dM/d\text{ln}(t)$ is the so-called magnetic viscosity.

**Figure 5:**
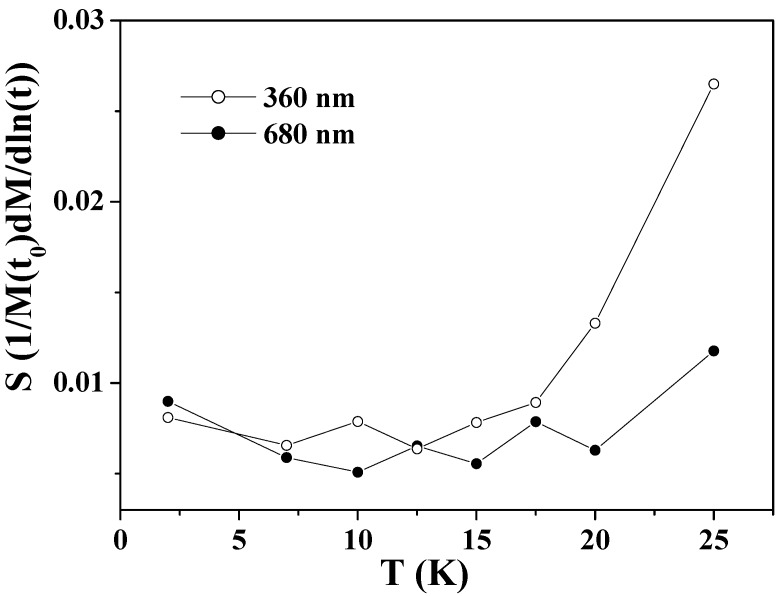
The magnetic viscosity *S* as a function of temperature for Co_3_O_4_ hollow spheres of 360 and 680 nm.

Figure 5 shows the obtained values of *S* as a function of temperature for the two samples. The *S* values of the larger hollow spheres depend little on temperature in the whole range below *T*_N_. The *S* values of the smaller hollow spheres exhibit nearly the same weak temperature-dependence as those of the larger ones below 15 K, but exhibit a significant increase above this temperature. This is consistent with the faster relaxation of the FC magnetization in the smaller particles at above 15 K as shown in Figure 3(b). The faster relaxation at above 15 K is known to be caused by the thermally activated relaxation [72,75]. For low temperatures 2 ≤ *T* < 15 K, the *S* values for the two samples show near-temperature-independence and they do not extrapolate to zero at 0 K. This phenomenon indicates the existence of relaxation processes other than the thermally activated one, as previously reported in Co_3_O_4_ nanoparticles [72] and ferritin protein [75].

### 4.2. Exchange Anisotropy

**Figure 6:**
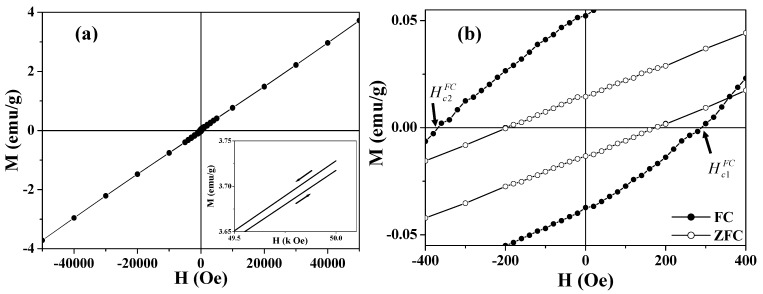
Hysteresis loops of 360 nm Co_3_O_4_ hollow spheres. (a) The FC hysteresis loop measured at 2 K; the inset shows the high field irreversibility of magnetization; (b) The central part of the ZFC (open circle) and FC (solid circle) hysteresis loops measured at 2 K.

Hysteresis loops measured after field cooling through the Néel temperature of AFM nanomaterials are usually used in exploring the exchange coupling between the surface UCM and the AFM core [65,66,67,68,69,70,72,73,74]. The FC hysteresis loop at 2 K for the 360 nm hollow spheres is presented in Figure 6. It is worthy noting that the FC loop opens up to 50 kOe (see the inset of Figure 6(a)), which indicates the existence of a high surface anisotropy layer [74]. The *M*-*H* curve is essentially linear up to 50 kOe except for the presence of ferromagnetism at lower fields. The ferromagnetic component appears to saturate around 5 kOe and is obtained by fitting the high field region with $M(H,T)=M_{0}(T)+\chi '(T)H$. The *M*_0_ values obtained after fitting the high field region are 0.036 and 0.068 emu/g for the 680 and 360 nm hollow spheres, respectively. The *M*_0_ value of the smaller spheres is nearly twice as large as that of the larger ones, which is consistent with the result of the FC magnetization measurements shown in Figure 3. 

Figure 6(b) shows the central portion of the FC loop in Figure 6(a). Compared with the zero-field-cooled (ZFC) loop, remarkably, the FC loops are broadened and are shifted toward negative magnetic fields, *i.e.*, $\left|H_{c2}^{FC}\right|>\left|H_{c1}^{FC}\right|$(see Figure 6(b)). The enhanced coercivity and the shift of the FC loop are generally known as exchange bias [65,66,67,68,69,70,72,73,74,76,77,78]. For the asymmetric FC loops, the coercive field and exchange bias field are defined as $H_{c}^{FC}=(H_{c1}^{FC}-H_{c2}^{FC})/2$ and $H_{eb}=(-H_{c2}^{FC}-H_{c1}^{FC})/2$, respectively [78]. The smaller hollow spheres (360 nm) show $H_{c}^{FC}$= 330 Oe and $H_{eb}$= 40 Oe, while the larger particles (680 nm) have $H_{c}^{FC}$= 175 Oe and $H_{eb}$= 20 Oe. The horizontal shift of the hysteresis loop usually occurs when a ferromagnet (FM) is in contact with an AFM, and the entire system is cooled below the Néel temperature of the AFM [76,77]. The exchange coupling present at the interface between the FM and the AFM induces a unidirectional anisotropy of the ferromagnetic layer. In the case of hollow magnets, the shell layer of the hollow sphere consists of AFM nanoparticles (~16 nm), each of which is coated with an FM layer derived from the UCM. The exchange bias was attributed to the exchange coupling between the surface FM (or UCM) layer and the AFM core of the nanoparticle. This proposed mechanism was supported by the fact that the sample with a larger UCM exhibits a larger exchange bias. 

As one conclusion from the FC magnetization experiments described in this section, the greatly enhanced UCM in the hollow spheres indicates that the pronounced curvature and the confinement of the thin shell layer of the hollow structures play a significant role in introducing the structural imperfection and lattice defects, in addition to the size effects. Therefore, it is expected to effectively tune the UCM and exchange bias by adjusting the diameter and shell thickness of the hollow spheres in the near future. 

## 5. Magnetization and Hysteresis Behaviors of Magnetite (Fe_3_O_4_) Hollow Spheres

Magnetite, which was discovered in loadstone in the 6th century B.C., was the first magnet known to humans. The mysterious powers of this black solid have fascinated people ever since. Magnetite has been utilized in tools and instruments such as compasses [79] and ferrofluids [80], and has attracted academic interest due to the Verwey transition, which is a metal-insulator transition caused by electron-electron correlation [81,82], and due to its half-metallic behavior with high Curie temperature (~860 K), which is applicable to spintronics [83,84]. Recently, extensive studies have been performed on nanostructured magnetite materials such as nanoparticles [85], nanowires and nanotubes [86,87], with an eye toward applications in magnetic separation, spintronics, and biomedical fields. 

Submicron-size magnetite hollow spheres have been synthesized by many groups. Yu *et al.* have prepared Fe_3_O_4_ hollow spheres (200–400 nm in diameter) with open pores (~30 nm) in shell layers using dodecylamine micelles as a template [51]. Zhu *et al.* reported the synthesis of single-crystal magnetite hollow spheres with a diameter of 200–300 nm by a template-free solvothermal route [53]. Furthermore, one-dimensional chainlike arrays of Fe_3_O_4_ hollow spheres with a diameter of 100–200 nm have recently been reported by Huang *et al.* [54]. However, most of the magnetite hollow spheres showed a large size distribution and there were no detailed studies on their magnetic properties, such as magnetization and hysteresis behaviors. Recently, Ye *et al.* have prepared magnetite hollow spheres with rather uniform size of outer diameter ca. 650 nm and shell thickness ca. 40 nm [26], and systematically studied their magnetic properties in comparison with magnetite dense particles (ca. 820 nm in diameter) [88] and ground nanoparticles (NPs) (~25 nm) obtained directly by grinding the submicron-size hollow spheres [26]. 

### 5.1. Verwey Transition

**Figure 7:**
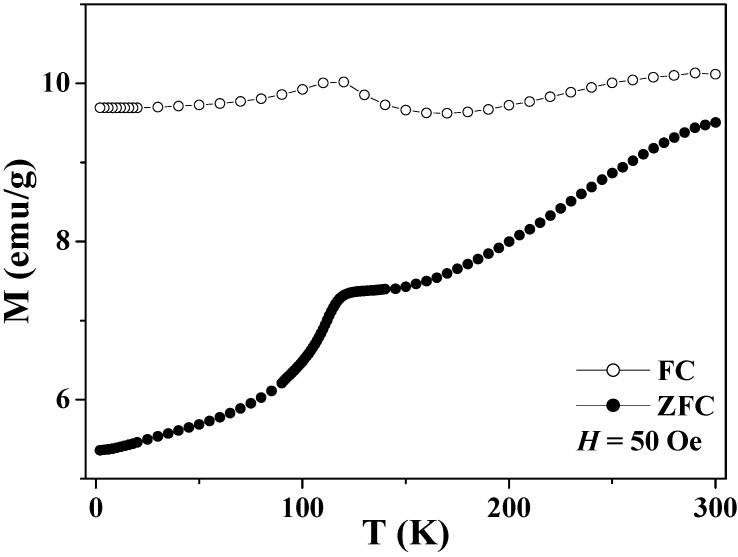
Temperature dependence of the zero-field cooled (ZFC) and field-cooled (FC) magnetizations of the magnetite hollow spheres.

Bulk magnetite undergoes the Verwey transition at around 120 K, and the charge ordering of the Fe^2+^ and Fe^3+^ ions on the octahedral sites has been proposed to account for the phase transition, although this mechanism is still under debate [81,82]. The Verwey transition leads to a change in conductivity by two orders of magnitude and also a significant drop in magnetization across the transition temperature. The Verwey transition has recently been reported in submicron-size magnetite hollow spheres by the ZFC-FC curve, as shown in Figure 7 [26]. Both the ZFC and FC curves peaked broadly at around 110 K, which was attributable to the Verwey transition [81,82,85]. The fact that the Verwey transition temperature was lower than that of bulk magnetite was probably due to the submicron diameters of the hollow spheres and the nanoscale sizes of the grains in the shell layer, since the ZFC curve of the ground NPs displayed the phase transition at a much lower temperature of 20 K [26]. In addition, the splitting of the ZFC and FC curves occurs in the whole temperature range of 2–300 K, indicating the blocking temperature of the hollow spheres is higher than 300 K.

### 5.2. Magnetization Curves and Hysteresis Behaviors

**Figure 8:**
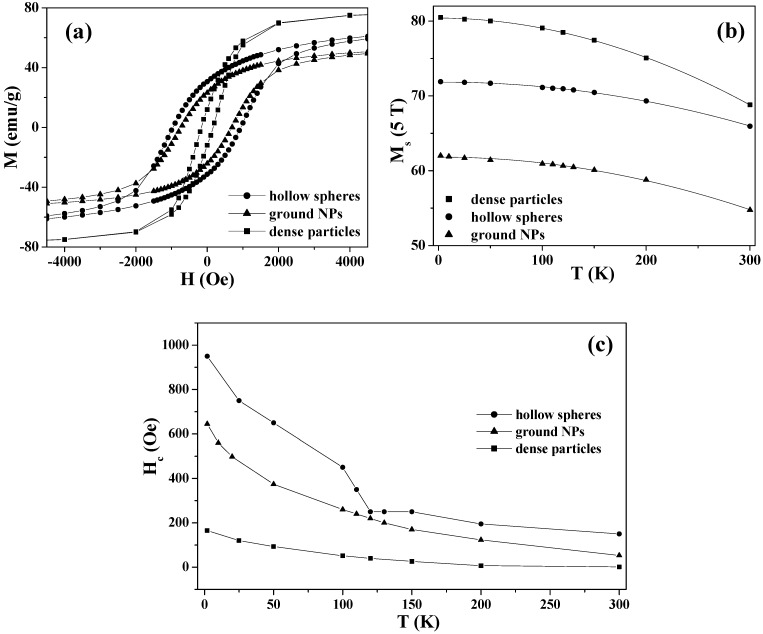
(a) *M*-*H* curves at 2 K, (b) *M*_s_ (5 T) *vs.* *T*, and (c) *H*_c_ *vs.* *T* for the magnetite hollow spheres, dense particles and ground NPs. The solid lines in (b) are the best fittings to the Bloch law, while the solid lines in (a) and (c) are the guidance to eye.

Magnetization curves (*i.e.*, *M*–*H* curves) and the hysteresis behaviors of magnetite hollow spheres were carefully studied in the temperature range of 2–300 K [26]. Figure 8(a) shows the results at 2 K in comparison with those of the magnetite ground NPs and dense particles. The hollow spheres exhibited the highest saturation remanence *M*_rs_, and possessed the largest maximum energy product, (*MH*)_max_, which corresponds to the area in the hysteresis loop. This result indicates that one way to achieve magnetically hard materials is by assembling soft nanoparticles into spherical hollow structures.

The saturation magnetizations (*M*_s_) of the magnetite hollow spheres, dense particles and ground NPs are significantly lower than that of bulk magnetite (97 emu/g), which is attributable to the surface disorder and/or spin canting at the surface of the particles [85,89]. Figure 8(b) shows the temperature dependences of the saturation magnetizations derived from the *M*-*H* curves measured at different temperatures. For the three samples, the *M*_s_ obtained at 5 T showed a steady decrease with increasing temperature, and the thermal decreases were fitted by the Bloch law [90]:

(2) $$M_{s}(T)=M_{s}(0)\left[1-B_{0}T^{n}\right]$$

where *M*_s_(0) is the saturation magnetization at zero temperature, *B*_0_ is the Bloch constant related to the exchange integral and *n* is the Bloch exponent. The best fitting results for the three samples are listed in Table 2 in comparison with the bulk magnetite reported in Ref. [90]. For all three samples, the Bloch exponents are very close to that of bulk magnetite (*n* = 2.0), but different from recent reports (*n* = 1.5) on magnetite nanoparticles [85] and nanowires [87]. Further, the Bloch constant of the hollow spheres is one order of magnitude smaller than those of the dense particles and ground NPs, which is consistent with the finding that the saturation magnetization of the hollow spheres has the weakest temperature dependence [Figure 8(b)]. 

Figure 8(c) shows the temperature dependence of the coercivities *H*_c_ for the three samples. As the temperature decreased from 300 to 2 K, the *H*_c_ of the hollow spheres increased from 150 to 950 Oe, with an anomaly at the Verwey transition. Below the transition temperature, *H*_c_ was enhanced, due to an increase in magnetocrystalline anisotropy accompanied by the structural change from cubic to monoclinic [82,85,91,92]. In contrast, the *H*_c_ values of the dense particles and ground NPs indicated a gradual increase with a decrease in temperature and the enhancements of *H*_c_ were less significant than that in the hollow spheres. 

**Table 2:** Saturation magnetization *M*_s_(0), Bloch constant *B*_0_, and Bloch exponent *n* for the magnetite hollow spheres, dense particles, ground NPs, and bulk magnetite.

Samples	*M*_s_(0)/emu g^-1^	*B*_0_/K^-*n*^	*n*
Hollow spheres	71.8	6.94 × 10^-7^	2.05 ± 0.04
Ground NPs	61.8	1.36 × 10^-6^	1.99 ± 0.06
Dense particles	80.4	2.20 × 10^-6^	1.95 ± 0.03
Bulk (Ref. 90)	97.0	5.54 × 10^-7^	2.0

### 5.3. Possible Domain States 

**Figure 9:**
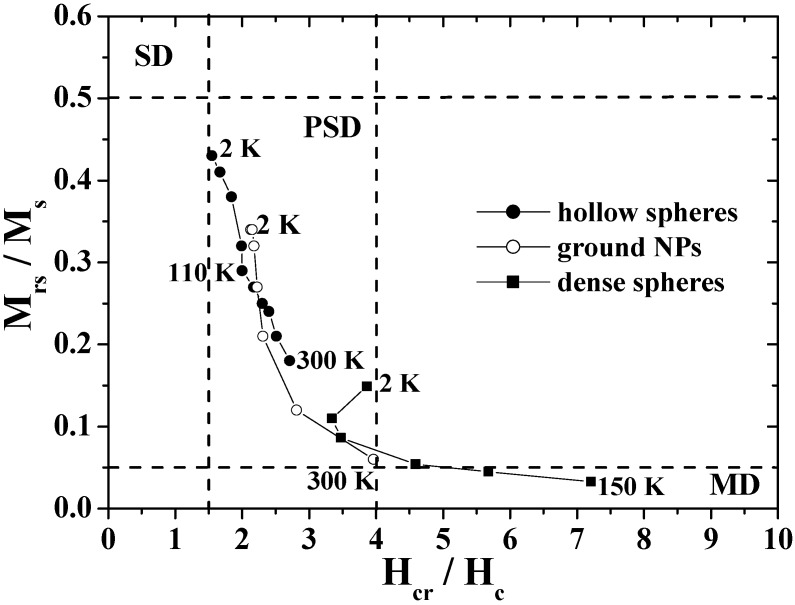
The Day plots of *M*_rs_/*M*_s_ versus *H*_cr_/*H*_c_ for the hollow spheres, dense particles and ground NPs.

The unique hysteresis behaviors and possible domain states were further elucidated by using a Day plot [26,91,92], in which the values of *M*_rs_/*M*_s_ are plotted as a function of *H*_cr_/*H*_c_, where *H*_cr_ is the reverse field required to reduce the remnent magnetization to zero [93,94]. Figure 9 shows the Day plots of *M*_rs_/*M*_s_ versus *H*_cr_/*H*_c_ for the hollow spheres, ground NPs, and dense particles in the temperature range of 2–300 K. In the figure, the single-domain (SD), pseudo-single-domain (PSD), and multi-domain (MD) states are separated from each other. The PSD state implies that the magnetic behavior is intermediate between SD and MD [91,92]. The plots for the dense particles are reasonably located in the MD region as are those for bulk magnetite [26], and the plots for the ground NPs move from the MD region to the center of the PSD region as the temperature decreases. However, the plots for hollow spheres appear in the PSD region with the characteristic temperature dependence; the plots approach the SD region with a decrease in temperature, particularly below the Verwey transition. The domain state of hollow spheres is closer in nature to SD than that of the ground NPs and dense particles, in spite of the submicron size. 

This feature of the hollow sphere was caused by its unique shape. In the case of a spherical hollow magnet with low magnetocrystalline anisotropy, the magnetization was theoretically predicted to predominantly follow the circumference in order to minimize the stray field, and hence the curling-vortex state with the vortex singularities on the poles of the hollow sphere was formed as a ground state [27,95,96,97]. The magnetizations in the hollow sphere were aligned with the field direction at the saturated field and were expected to form the onion state just below the saturation field. The switching between the curling vortex and onion states resulted in a magnetization jump as demonstrated in submicron-size ferromagnetic rings [5,95,96,97]. As a result, the coercivity was enhanced in the magnetite hollow spheres, which brought about a decrease in the value of *H*_cr_/*H*_c_, resulting in the Day plots for the hollow spheres appeared to indicate the SD state.

### 5.4. Magnetic Anisotropy of Thin Films

**Figure 10:**
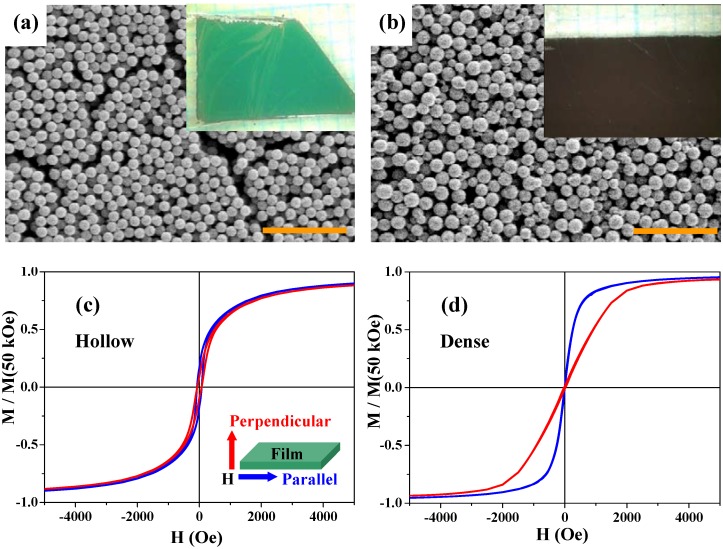
SEM images of thin films of the hollow (a) and dense (b) spheres. The scale bars are 5 μm in (a) and (b). The insets in (a) and (b) are photographs of the corresponding films. (c) and (d) are *M*-*H* curves at 300 K for the thin films of the hollow spheres and the dense particles, respectively, with magnetic field *H* parallel (blue curves) and perpendicular (red curves) to the film plane (see the inset of (c)). Magnetizations are normalized by the value at 50 kOe.

Magnetite is a low magnetocrystalline-anisotropy material, and its thin films usually exhibit two-dimensional (2D) shape anisotropy; the parallel magnetization is larger than the perpendicular one, reflecting dipole-dipole interactions in the films [98]. Recently, multilayer thin films (~3–5 μm in thickness) of magnetite hollow spheres were prepared and their magnetic anisotropy properties were studied [47]. First, thin films of the precursor particles (*i.e.*, iron-salt/PS particles) were fabricated by the fluidic-cell method [47,99]. And then the thin films of magnetite hollow spheres were obtained after calcinations. For comparison, multilayer thin films of the dense particles were directly prepared by the fluid-cell method from aqueous solution of the magnetite dense particles. Figure 10(a) and Figure 10(b) show scanning electron microscope (SEM) images and photographs (inset) for the two films; both the hollow and dense particles are rather densely packed in the films and there are no essential differences in packing and surface morphology between the two samples.

The *M*-*H* curves of the two films were measured under magnetic fields parallel and perpendicular to the 2D films. Figure 10(c) and Figure 10(d) show the results at 300 K for the hollow and dense particles, respectively. The blue and red curves indicate the magnetizations parallel and perpendicular to the 2D layer, respectively. While the multilayer thin films of the dense particles clearly exhibit the magnetic anisotropy especially below 5 kOe [Figure 10(d)], as expected from the usual magnetite thin films, the parallel and perpendicular magnetization curves for the hollow spheres are superimposed [Figure 10(c)], indicating there is no magnetic anisotropy in this thin film. The anisotropy of the dense spheres was caused by the interparticle magnetic dipole-dipole interactions, since there was no magnetic anisotropy in the one-particle magnetization. Since the hollow spheres are low spin-density magnets, such magnetic interactions between the hollow magnets are much weaker than those in the dense particles. Therefore, the thin films of hollow spheres exhibited isotropic magnetizations. This result indicates that the hollow spheres may be used in an advanced procedure for the suppression of magnetic anisotropy.

As shown in this section, the magnetite hollow spheres exhibited unique magnetic properties, such as high blocking temperature, enhanced coercivity and curling-vortex magnetization state, as well as magnetic isotropy. These results suggest that the magnetic hollow structures may have advantages for investigating the dynamic behaviors of spin rotation and relaxation by applying magnetic or electrical fields, and therefore have potential applications in spintronics, in which the aim is to achieve a spin-polarized current or current-control magnetization [7].

## 6. Optical Properties of Fe_3_O_4_ and Co_3_O_4_ Hollow Spheres 

**Figure 11:**
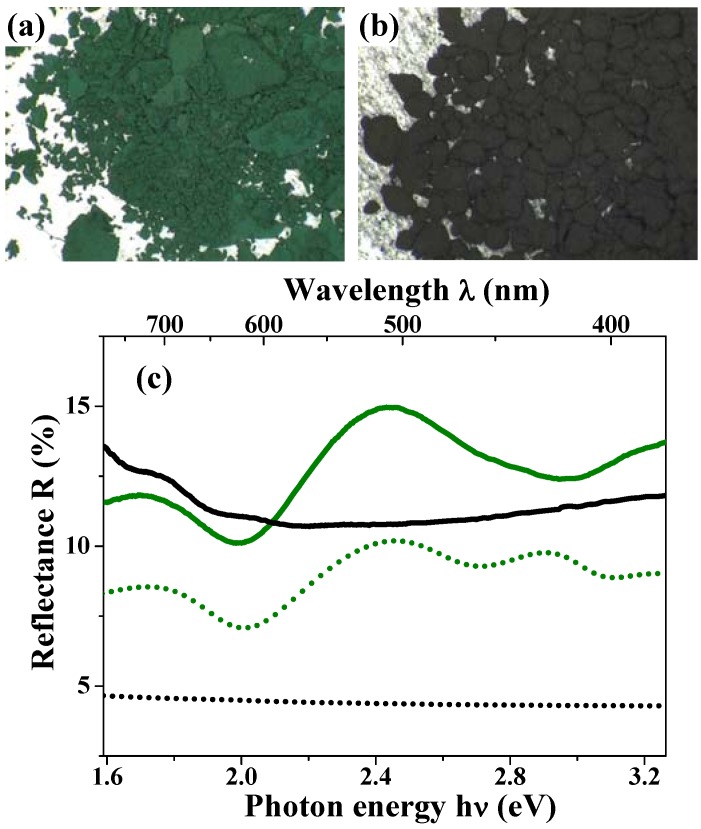
(a) and (b) are photographs of the hollow and dense particles, respectively; (c) Diffuse reflectance spectra for the magnetite hollow spheres (green solid curve) and the dense particles (black solid curve). The calculated reflectance spectra based on the theory of Mie scattering for the hollow spheres (green dotted curve) and the dense particles (black dotted curve) reproduce the peak positions of the experimental data. The absolute experimental values are typically not reproduced by the calculations, due to the approximation of the Kubelka-Munk approach.

Hollow structures and submicron sizes induce not only unique magnetic properties but also anomalous optical properties. Figure 11(a) and Figure 11(b) show the photographs for the magnetite hollow and dense particles [47]. The magnetite hollow spheres look green; this color is thoroughly different from the black color of the dense particles, which have a spherical shape and size similar to those of the hollow spheres. Magnetite materials always appear black in the bulk solids [88] and even as nanoparticles [85].

The green solid curve in Figure 11(c) shows the diffuse reflectance spectra for the hollow spheres. This curve indicates an obvious peak at around 510 nm, which corresponds to the wavelength of the green light. The black solid curve in this figure depicts the results for the dense particles; it is featureless and there is no peak at around 510 nm. These results are well consistent with the appearances of the two materials.

In general, color results from the interactions between light and matter, namely absorption, scattering and reflection [100]. These are strongly dependent on the shape and size of the substance. When the size of the substance is comparable to the wavelength of incident light, Mie scattering occurs [100]. The green color of the magnetite hollow spheres was interpreted in terms of Mie scattering because the submicron size of the particles was comparable to the wavelength of visible light. The reflectance *R* for the highly aggregated particles was theoretically calculated through the Kubelka-Munk approach [101]:

(3) $$R=1+\frac{K}{S}-{\left(\frac{K^{2}}{S^{2}}+\frac{2K}{S}\right)}^{1/2}$$

with $K=\frac{3Q_{abs}}{2r_{2}}$ and $S=\frac{9Q_{sca}(1-\bar{\text{cos}\theta })}{16r_{2}}$, where the absorption efficiency *Q*_abs_, the scattering efficiency *Q*_sca_, and the asymmetric factor $\bar{\text{cos}\theta }$ for a single particle were obtained based on the Mie scattering theory [100,102]. In the simulations, the hollow sphere was treated as a core-shell structure consisting of an ambient core with radius *r*_1_ and refractive index *m*_1_ and a magnetite shell with radius *r*_2_ and refractive index *m*_2_ [47]. The green dotted curve in Figure 11(c) is the theoretical best fit to the experimental curve for the hollow spheres with the following parameters: *m*_1_ = 1.0 (fixed), *m*_2_ = 2.55 + 0.70i, *r*_1_ = 285 nm (fixed), and *r*_2_ = 325 nm (fixed). The obtained *m*_2_ values are consistent with the reported values for bulk magnetite [103,104,105]. The theoretical curve reproduces the weak reflectance peak at around 725 nm and the higher one at 510 nm. The extra peak at 425 nm in the simulation disappeared in the experimental curve, presumably due to the size distribution and/or the presence of partially broken particles in the sample. 

For the dense particles which are homogeneous media, the simulation of the reflectance spectra was carried out based on Eq. 3 and the Mie scattering theory by setting *m*_1_ = *m*_2_ = *m*. The black dotted curve in Figure 11(c) shows the results of this simulation with the refractive index *m* = 2.55 + 0.70i, which is the value optimized above, and the particle radius *r*_2_ = 410 nm. The theoretical simulation well reproduces the weak wavelength dependence of diffusion reflectance of the dense particles. Although the dense particles had a noticeable size distribution, the calculated reflectance spectra did not show large differences by setting the radius *r*_2_ from 250 to 450 nm. It is notable that this feature is caused mainly by the light absorption in the visible range, due to the dense structure; the absorption of the hollow spheres is much weaker than that of the dense particle, due to its low-density structure [Figure 1(d)]. 

**Figure 12:**
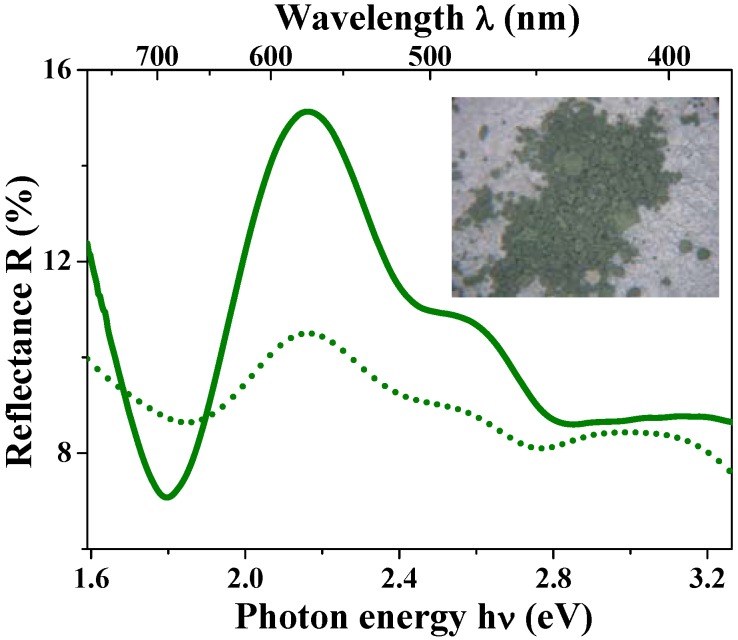
Diffusion reflectance spectra for Co_3_O_4_ hollow spheres. The green solid and dotted curves represent experimental and simulation data, respectively. The inset photograph shows the light green color of the Co_3_O_4_ hollow spheres.

The anomalous optical properties caused by the low-density hollow structures were also observed in Co_3_O_4_ hollow spheres. The Co_3_O_4_ hollow spheres with outer diameter of 760 nm and shell thickness of 70 nm displayed a light green color (see the photograph in the inset of Figure 12), which was in contrast with the usual black color of bulk Co_3_O_4_. The reflectance spectra of Figure 12 exhibit a main reflectance peak at around 575 nm, which is consistent with the color appearance. By setting the Co_3_O_4_ hollow sphere as a ambient-core/Co_3_O_4_-shell structure and fixing the parameters *m*_1_ = 1.0, *r*_1_ = 310 nm, and *r*_2_ = 380 nm in the theoretical simulation, the reflectance spectra were reproduced (dotted line in Figure 12) and the optimized refractive index of *m*_2_ = 1.88 + 0.20i was close to the values in bulk [105] and/or thin films of Co_3_O_4_ [106]. 

For both the hollow spheres of Fe_3_O_4_ and Co_3_O_4_, the green color disappeared when the hollow structures were destroyed by grinding. Therefore, it is concluded that the green color of the hollow spheres is caused by the Mie scattering, as an inevitable property of the uniform submicron sizes of the particles and the peculiar low-density hollow structures. The unusual green color may lead to a promising way to design novel magneto-optical functions of magnetic materials, such as wavelength specific Kerr and/or Faraday effects.

## 7. Conclusions and Outlook 

We have reported on recent progress in the study of submicron-size magnetic hollow spheres. The hollow spheres with uniform size and morphology were fabricated through two steps: first, inorganic precursor materials were homogeneously deposited on the surfaces of PS beads; then various phases (ccp- and hcp-Co, Co_3_O_4_, α-Fe, Fe_3_O_4_, and α-Fe_2_O_3_) of hollow spheres were obtained by selectively optimizing the calcination conditions. The synthetic strategy showed advantages for obtaining a uniform outer diameter and shell thickness of hollow spheres, which enabled people to achieve and tune amomalous physical properties. Futher, it is noteworthy that the ccp and hcp phases of cobalt could be separated and a single phase of hcp-Co with high magnetic anisotropy could be selectively synthesized using the synthetic method. The hollow spheres exhibited significantly different physical properties from those of flat thin films and solid counterparters. Weak ferromagnetism arising from uncompensated spins and exchange anisotropy were found in the antiferromagnetic Co_3_O_4_ hollow spheres. Magnetite hollow spheres showed significantly enhanced coercivity and an unusual green color. These unique physical properties were interpreted in terms of the effects of the hollow structures, curved surfaces, and submicron sizes, in comparison with submicron-size dense particles and nanoparticles. These results indicate that the spherical hollow magnets may have potential applications in data-storage media, spintronics, and magneto-optical devices. However, some fundamental aspects need to be further studied, such as direct observation on magnetic domain structures, electrical and magnetic transport on individual magnetic hollow spheres, and magneto-optical properties.

Further, spherical hollow structures are advantageous for various surface modifications. Ohnish *et al.* have recently carried out chemical modifications on the surfaces of the magnetite hollow spheres by grafting poly(3-*N*-methylvinylpyrdinium) macromolecules via polymerization of 4-Vinylpyridine and *N*-alkylation reactions [30]. After the surface modification, the particles exhibit high and stable dispersibility in water for a few hours, while maintaining their hollow structures and magnetic properties. The surface modification may open up possible applications of the spherical hollow magnets in the areas of catalysis, ferrofluids, drug-deliver systems, and magnetic photonic crystals. 

In addition to surface modifications, the interior hollow pore of the hollow spheres may be constructed into multilevel architectures, such as a core-in-hollow-shell (or rattle-type) structure, multishell structure and multichamber structure [11,57,58]. Such complicated hollow architectures have demonstrated a wealth of optimized properties in many nonmagnetic materials. For instance, tin nanoparticle-encapsulated elastic hollow carbon spheres (TNHCs) rattle-structured nanomaterials show high performance as anode materials in lithium-ion batteries [107], and multishell Cu_2_O hollow microspheres show greatly improved sensitivity to ethanol gas [108]. However, there are few reports on the multilevel interior structures in magnetic hollow spheres. If a magnetic rattle-type hollow structure consisting of antiferromagnetic and ferromagnetic materials as the core and hollow shell could be constructed, it might be possible to controllably adjust the exchange coupling between the AFM and FM as well as the exchange anisotropy by applying external magnetic fields. Moreover, the multilevel magnetic hollow spheres may exhibit multifunctions through the incorporation of functional materials (e.g., semiconductors or fluorescent materials) into the multilevel architectures. These features make the magnetic hollow structures unique and promising candidates as advanced magnetic materials.
